# Determinants of the European Sovereign Debt Crisis: Application of Logit, Panel Markov Regime Switching Model and Self Organizing Maps

**DOI:** 10.3390/e25071032

**Published:** 2023-07-08

**Authors:** Jean-Pierre Allegret, Raif Cergibozan

**Affiliations:** 1 CNRS, GREDEG, Bâtiment 2, Campus Azur du CNRS, Université Côte d’Azur, 250 rue Albert Einstein, CS 10269, F-06905 Sophia Antipolis Cedex, France; jean-pierre.allegret@univ-cotedazur.fr; 2 Department of Economics, Kirklareli University, Kayali Kampüsü B-Blok, 39000 Kirklareli, Turkey

**Keywords:** European debt crisis, fiscal stress index, logit, Markov regime switching model, self-organizing maps

## Abstract

The study aims to empirically identify the determinants of the debt crisis that occurred within the framework of 15 core EU member countries (EU-15). Contrary to previous empirical studies that tend to use event-based crisis indicators, our study develops a continuous fiscal stress index to identify the debt crises in the EU-15 and employs three different estimation techniques, namely self-organizing map, multivariate logit and panel Markov regime switching models. Our estimation results show first that the study correctly identifies the time and the length of the debt crisis in each EU-15-member country. Empirical results then indicate, via three different models, that the debt crisis in the EU-15 is the consequence of deterioration of both financial and macroeconomic variables such as nonperforming loans over total loans, GDP growth, unemployment rates, primary balance over GDP, and cyclically adjusted balance over GDP. Furthermore, variables measuring governance quality, such as voice and accountability, regulatory quality, and government effectiveness, also play a significant role in the emergence and the duration of the debt crisis in the EU-15.

## 1. Introduction

Over the last decade, the European Union went through the most severe economic and political crisis since its creation following World War II. Some economists (i.e., [1,2,3,4,5,6,7]) stated that the crisis was the result of contagion of the US subprime crisis to Europe: as the crisis spread to Europe, governments and central banks heavily intervened in real and financial sectors to limit the negative impacts of the crisis. These expansionary policies and bank rescue plans (in other words, nationalization of private debt) resulted in a dramatic rise in public debt stock, leading then to a sovereign debt crisis in some Eurozone member countries.

Some argued that the crisis was related to increasing fiscal deficits and rising public debt stock, but these problems are the consequences of the structural factors associated with the Eurozone (i.e., [8,9,10,11,12,13,14]). The main argument here is the Eurozone is not an optimum currency area a la Mundell [15], since there is no risk sharing system such as an automatic fiscal transfer mechanism to redistribute money to areas/sectors which have been adversely affected by the capital and labor mobility. Moreover, Eurozone is a monetary union without a fiscal union: this design, permitting the free riding of fiscal policies within a framework of common monetary policy, led to differences in inflation rates within the Eurozone member countries. Inflation differences in turn caused a decrease in the trade competitiveness of high-inflation countries, i.e., Greece, Spain. As the option of improving the competitiveness of the economy through exchange rate depreciation was not available, because of the common currency, trade deficits steadily rose in the Southern peripheral countries, leading to constant increases in public debt stock [16]. This was not an important problem until the outbreak of the global financial crisis. With the transition to European Monetary Union (EMU), increasing capital inflows towards peripheral countries resulted in low interest rates facilitating the rollover of the debt stock. In addition, low interest rates led to a decrease in household savings and increased consumption, causing external deficits and an increase in private debt stock. 

This study aims to empirically identify the determinants of the European debt crisis. To do so, we employ three different estimation techniques, namely SOM, logit, and Markov models. The main reason to use different methods is the fact that using different methodologies have led to inconsistent results in terms of crisis determinants and crisis prediction (see [17,18,19,20] for further discussion). Hence, we first apply the SOM approach, which allows us to visualize, via crisis maps created for each country, the transition from noncrisis to crisis states. Furthermore, the SOM analysis gives us variables’ order of importance in explaining the occurrence of the debt crises in the EU member countries. In other words, the SOM analysis serves as a filter to determine which indicators should be included into the logit and Markov model estimations. Then, we estimate logit and Markov models with the variables found to be significant by the SOM approach.

This paper brings empirical contributions to the literature on fiscal stress in a monetary union [21,22,23,24]. In the first step, we identify and date debt crises by defining a new fiscal stress index. Second, we use a large data set composed of 51 leading indicators to explain the European debt crises. In particular, this paper includes an important number of governance indicators that have largely been ignored in explaining debt crises. Third, we use different econometric tools—namely the self-organizing maps (SOM), the multivariate logit model (MLM), and the panel Markov regime switching model (PMRSM)—to identify the determinants of the European debt crisis. Our study therefore offers the opportunity of a comparative analysis between different model estimations, which has not been conducted yet in the literature. 

According to the results, in addition to financial and macroeconomic variables, such as nonperforming loans over total loans, GDP growth, primary balance over GDP, unemployment, and cyclically adjusted balance over GDP, governance variables (i.e., voice and accountability, regulatory quality and government effectiveness) also play a significant role in the emergence of the European debt crisis. In addition, forecast performances estimates suggest that our different models perform relatively well to predict the debt crisis in the Eurozone.

The remainder of the paper is organized as follows. Section 2 presents data and methodology and the definition of our fiscal stress index. Section 3 discusses estimation results. Section 4 concludes.

## 2. Data and Methodology

### 2.1. The Definition of Fiscal Stress Index

Debt crises are usually identified and dated by a combination of events, such as the inability of borrowers to pay the interest or principal on time, large arrears, or large IMF loans to help the borrower avoid a default. In other words, dating debt crises is generally event-based and is typically founded on the available ex post figures (i.e., [21,25,26]). However, this dating method has several shortcomings. It is based primarily on information about government actions undertaken in response to fiscal stress and depend on information obtained from regulators and international organizations or rating agencies. In addition, the events method identifies crises only when they are severe enough to trigger market events; crises successfully contained by prompt corrective policies are neglected. This means that empirical work suffers from a selection bias. Therefore, in order to fulfill these shortcomings, we develop a fiscal stress index like currency crisis indictors, a la Eichengreen et al. [27] or Kaminsky and Reinhart [28], in order to identify the dates of debt crisis episodes occurred in EU-15 countries over the period from 2003–2015.

The data used for constructing the fiscal stress index (FSI) are gathered from Oxford Economics and IMF International Financial Statistics for the period from 2003–2015. Our fiscal stress index is defined as a continuous variable rather than event-based, contrary to previous studies. The bond yield pressure, imputed interest rate on general government debt minus the real GDP growth rate, public sector borrowing requirements, general government gross debt, and cyclically adjusted primary balance variables are used in calculating our fiscal stress index. The selection of variables in the construction of the index is based on Baldacci et al. [21], McHugh et al. [26], and Hernandez de Cos et al. [23]. Note also that the variables are standardized or weighted according to the empirical crisis literature. The weights of the components of the crisis index are chosen to equalize their volatility and thus avoid the possibility of one of the components dominating the index, allowing us to obtain consistent results concerning dates of debt crises.

The fiscal stress index is calculated as follows:

(1) $$FSI_{i,t}=\frac{\Delta BYP_{i,t}}{\sigma_{BYP_{i,t}}}+\frac{\Delta {\left(r-g\right)}_{i,t}}{\sigma_{{\left(r-g\right)}_{i,t}}}+\frac{\Delta PSBR_{i,t}}{\sigma_{PSBR_{i,t}}}+\frac{\Delta GGGD_{i,t}}{\sigma_{GGGD_{i,t}}}-\frac{\Delta CAPB_{i,t}}{\sigma_{CAPB_{i,t}}}$$

where *BYP* (bond yield pressure) is government bond spreads (relative to 10-year US Treasury bonds), *r − g* is the imputed interest rate on general government debt minus real GDP growth rate, *GGGD* is general government gross debt, and *CAPB* indicates cyclically adjusted primary balance/GDP. Sub-indexes represent *t* as time, *i* as country, and Δ is the differential operator. Increases in *BYP*, *r − g*, *PSBR*, and *GGDD* augment fiscal pressure, while increases in *CAPB* reduce fiscal pressure. Because increases in *CAPB* indicate a balanced budget, its effect is expected to be negative.

We define a debt crisis hitting country *i* at time *t*, *C_i,t_*, as a binary variable that can assume either 1 (when the FSI is above its threshold value) or 0 (otherwise):

(2) $$C_{i,t}=\left\{ \begin{array}{l} 1{\;\mathrm{if}\;\mathrm{FSI}}_{i,t}>\mathrm{optimal}\;\mathrm{threshold}\;\\ 0\;\mathrm{otherwise} \end{array}\right.$$

A critical point is to choose an ‘optimal’ threshold value. Several papers determine an arbitrary threshold. The higher the threshold level is, the lower the number of detected crises is, and vice versa. Therefore, this arbitrary threshold method results in different numbers and effective dates of crises as empirically shown by Kamin et al. [29], Edison [30], and Lestano and Jacobs [31] in the case of currency crises. 

In order to avoid problems related to threshold level, we consider different methods based on Candelon et al. [32] to determine the optimal threshold value for the fiscal stress index of each EU-15 country. For this purpose, we use accuracy measures, sensitivity-specificity graphics, and the KLR cut-off method Kaminsky et al. [33] to select the optimal threshold. In this study, we present two different cut-off values, country-specific and global, in the KLR cut-off method. The country-specific cut-off value is the cut-off value determined according to the country’s own fiscal stress index, while the global cut-off value is the cut-off value obtained from the fiscal stress index of all EU-15 countries.

The fiscal stress index for each EU-15 country is constructed according to the Equation (1). In order to identify debt crisis periods, we need to determine optimal threshold (cut-off) values, which are calculated using three different methods (see Table 1). Bold numbers indicate the optimal cut-off values for each country. 

**Figure 1:**
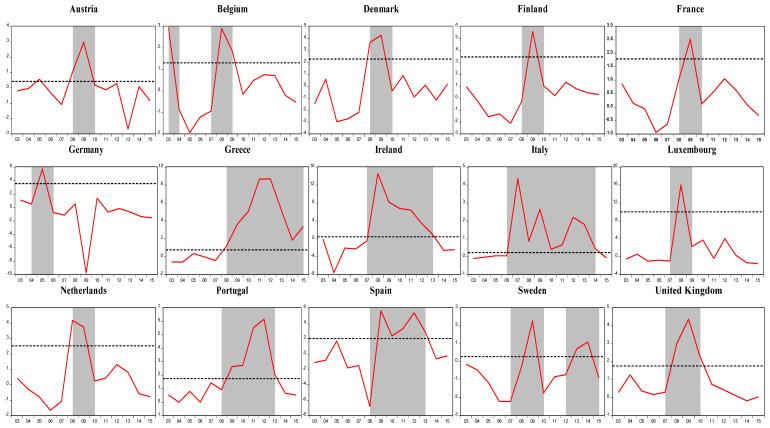
Fiscal stress indexes and their threshold values for EU-15 countries. **Note:** dashed areas indicate crisis periods.

Figure 1 presents the crisis and noncrisis periods for EU-15 countries: shaded zones indicate crisis periods, in other words, the period where the index value exceeds the optimal threshold value. As clearly seen from Figure 1, all EU-15 countries except for Germany seem to have gone through the debt crisis following the global financial crisis. As expected, the debt crisis in Greece, Ireland, Spain, the United Kingdom, Italy, and Portugal seem to have lasted longer compared to other countries. In addition, Greece seems to have not fully recovered from the debt crisis by the end of 2015.

When we compare our results with those of previous literature [21,22,23], we observe that they do not find any crisis episode in the cases of Austria, Belgium, Finland, France, and the Netherlands in the post-2003 period (see Table 2). Our fiscal stress index identifies more ‘debt crisis’ episodes than previous empirical studies applied to debt crises, since it measures the pressure or stress level in a country contrary to other fiscal stress definitions that focus mainly on default events. On the contrary, our results show that Austria in 2009, Belgium in 2003, 2008, and 2009, Finland in 2009, France in 2009, and the Netherlands in 2008 and 2009 had severe fiscal problems. Furthermore, Hernandez de Cos et al. [23] state that Greece, Ireland, Italy, and Portugal had a debt crisis from 2008 to 2010, while our index indicates that Greece from 2008 to 2015, Ireland from 2008 to 2013, Italy from 2007 to 2014, and Portugal from 2009 to 2013 suffered a debt crisis.

### 2.2. Leading Indicators

Our dataset consists of 51 leading indicators. The selection of leading indicators is based on the studies by Manasse et al. [34], Baldacci et al. [21], McHugh et al. [26], Berti et al. [22], and Hernandez de Cos et al. [23]. Table 3 presents definitions, sources, and descriptive statistics for the selected leading indicators used in the study. We consider five sets of indicators. The first set consists of public and real sector variables: GDP, inflation, unemployment, government expenditure/GDP, primary balance/GDP, cyclically adjusted balance/GDP, revenue/GDP, interest payments/revenue, interest payments/expenses, cash surplus/GDP, REER, savings/expenditures, tax revenue/GDP, and wages. The second category includes financial indicators that exert an influence on sovereign debt situations: bank capital/asset, nonperforming loans/total loans, banking sector leverage, M2/GDP, and banking crisis index. The study uses Laeven and Valencia’s [35,36] definition of a banking crisis.

Our third set of indicators encompasses different debt ratios: external debt/export, external debt/GDP, external debt government/GDP, external debt private/GDP, net debt/GDP, and household debt/GDP. Social indicators constitute our fourth set: health expenditure/GDP, public health expenditure/GDP, Gini coefficient, gross enrollment ratio, fertility rate, and age dependency ratio. Excessive increases in health expenditures, a deterioration in income distribution, a decline in education level and in fertility rate, and an increase in age dependency ratio are expected to increase the likelihood of a debt crisis.

Finally, our fifth and last set includes governance indicators. Only a very small number of studies have examined the effect of governance quality on the likelihood of debt crises [34,37]. In our study, unlike these studies, we directly use a large number of governance indicators in our model, including political stability risk rating, credit rating, trade-credit risk rating, government effectiveness, political stability and freedom from violence/terrorism, regulatory quality, rule of law, and voice and accountability variables. The deterioration of countries’ governance indicators is expected to increase the likelihood of a debt crisis. We use Kaufmann et al. [38] for defining governance indicators. Accordingly, voice and accountability cover freedom of expression, freedom of association, election of government, and free media for a nation’s citizens. Political stability and the freedom from violence/terrorism demonstrate the possibility of government destabilization or overthrow through unconstitutional political violence or terrorism. The government effectiveness indicator is the government’s policymaking and implementation quality and the credibility of its commitment to such policies, as well as the degree to which public services are independent of political repression. Rule of law shows the implementation of contracts in addition to opportunities for crime and violence; the quality of the police, courts, and property rights; and the level of trust and compliance of individuals with society. Control of corruption refers to the use of public power for special gains, with small or large corruption in addition to elite and private interests seizing public power. Political stability refers to the stability of the current government and the entire political system. Trade-credit risk rating means that the trading partner cannot fulfill its obligations. The democracy index refers to the country’s level of democracy.

### 2.3. Methodology

The previous literature testing the likelihood of a debt crisis rests on models such as logit-probit, signal approach, and Markov regime switching. We take a different approach by using three different methods in a comparative perspective. SOM or Kohonen maps (SOM model is a learning methodology introduced in the artificial neural network literature by Kohonen [39]), multivariate logit model (MLM), and panel Markov regime switching model (PMRSM). In addition, we test the stability of estimates. Last but not least, the predicting performance of each method is presented.

The SOM is a nonlinear and nonparametric method used to analyze high-dimensional datasets. Specifically, this model portrays low-dimensional images of high-dimensional data. An important contribution of this method compared to many econometric tools is that it does not rely on rigid assumptions. For instance, including too many variables at the same time may induce multicollinearity, where too many parameters cannot be predicted due to observation constraints. Although the SOM method has been used extensively in a large number of scientific fields since it first appeared in the literature, its use in economics is very rare (See [40,41,42,43,44,45,46,47,48,49]). For crisis literature, see Sarlin [50,51] and Sarlin and Marghescu [52].

A drawback of the SOM method is to interpret its components without specifying any definite relationship. In order to deal with this drawback, different approaches allow the identification of the significance of variables in SOM analysis. These approaches, which originate from the natural sciences, estimate different indexes such as the structuring index (SI), the relative importance index (RI), the cluster description index (CD), and the Spearman rank correlation index (SRC) [53].

The SI index has been originally developed by Park et al. [54] and Tison et al. [55,56]. A variable with a low SI value indicates that its effect on the cluster of the SOM map is low. In contrast, variables with high *SI* values explain a significant portion of the differentiation between cluster groups. The *SI* value of variable *i* is calculated as follows:

(3) $$SI_{i}=\sum_{j=1}^{S}\sum_{k=1}^{j-1}\frac{\left|w_{ij}-w_{ik}\right|}{\left\|r_{j}-r_{k}\right\|}$$

where the nominator and denominator show the weight and topological differences between *j* and *k* map units, respectively, while *S* represents the total number of map units.

In RI indexes, each variable is expressed based on the distance matrix as a pie chart proportional to the sum of the variables. In addition, the sum of these effects is standardized at 100. In other words, the importance of the variables in the model depends on the size they have in the pie chart. Accordingly, *i* is expected to have a high RI value if it is to have a high effect on the SOM structure.

Vesanto [57] uses the CD index, which expresses the variation in each cluster. Thanks to the CD index, the internal properties of each cluster can be displayed. The CD index is calculated as follows:

(4) $$CC_{i}=\sum_{l=1}^{C}S_{li}^{D}=\sum_{l=1}^{C}\frac{(C-1)S_{li}^{C}}{\sum_{m=1,m\neq 1}^{C}S_{mi}^{C}}\;\mathrm{where}\;S_{li}^{C}=\frac{\sigma_{li}}{\sigma_{i}}$$

where *σ_li_* and *σ_i_* indicate the standard deviations of the variable in cluster *l* and the whole data set, respectively, while *C* shows the total number of clusters. A high CD value calculated for a variable means that the variable has high significance when it occurs in different clusters.

These methods can give quite a different order of importance in estimates. Hence, in order to deal with this potential inconsistency, not only do we estimate the previous indexes, but we also estimate two different overall indexes to avoid any contradictory results. The overall index (1) is calculated with the following steps. First, four different index values are converted into percentage values. For this, the highest value of each index is accepted as 100 and all other values are calculated based on this value. The main purpose of doing this is to provide a chance to compare different indexes from the same unit. Second, as each index is expressed as a percentage, the following calculation is made so that each index has an overall weight equal to:

(5) $$Overall\;Index\left(1\right)=\frac{X_{i}\left(SI\right)+X_{i}\left(RI\right)+X_{i}\left(CD\right)+X_{i}\left(SRC\right)}{4}$$

where *X_i_* represents the *SI*, *RI*, *CD* and *SRC* values of the variable *i*.

The overall index (2) is calculated as follows:

$$Overall\;Index\left(2\right)=\frac{SI-\mu_{SI}}{\sigma_{SI}}+\frac{RI-\mu_{RI}}{\sigma_{RI}}+\frac{CD-\mu_{CD}}{\sigma_{CD}}+\frac{SRC-\mu_{SRC}}{\sigma_{SRC}}$$

where $\sigma_{SI}$, $\sigma_{RI}$, $\sigma_{CD}$, and $\sigma_{SRC}$ show the standard deviations for the *SI*, *RI*, *CD*, and *SRC* indexes, respectively. $\mu_{SI}$, $\mu_{RI}$, $\mu_{CD}$, and $\mu_{SRC}$ indicate the means of the *SI*, *RI*, *CD*, and *SRC* indexes, respectively. In the overall index (2), we subtract the value of each index by its means and then divide the result by its standard deviation in order to standardize the indices and ensure that no factor dominates the overall index. The influence of extreme results is minimized with the aim of obtaining more consistent results. In addition, the consistency of the indexes was checked via factor analysis and the results were found to be consistent.

Logit-probit models are widely used in debt crisis literature (e.g., [25,34,58,59]). In such models, the dependent variable, i.e., the fiscal stress index, is converted into a binary variable. It has a value of “1” for values above the threshold (signaling debt crisis periods) and “0” otherwise (normal periods).

The Markov model is also frequently used in papers on financial crises (i.e., [32,60,61,62,63]). The Markov model uses the crisis index in a continuous format. As a result, unlike the logit model, no information is lost regarding crisis duration. Specifically, the Markov model does not require a prior dating of crises; instead, identifying crisis periods are determined within the model itself [64]. In our estimation results, the Davies test also indicates the number of regimes chosen to be appropriate for the predicted models. As in the case of Abiad [64], Alvarez-Plata and Schrooten [62], and Lopes and Nunes [65], who used the Markov model for crises, our study also assumes two different regime periods. The period with lower mean and volatility indicates the tranquil or no crisis regime, while the second regime with higher mean and volatility is said to be crisis.

## 3. Estimation Results

**Figure 2:**
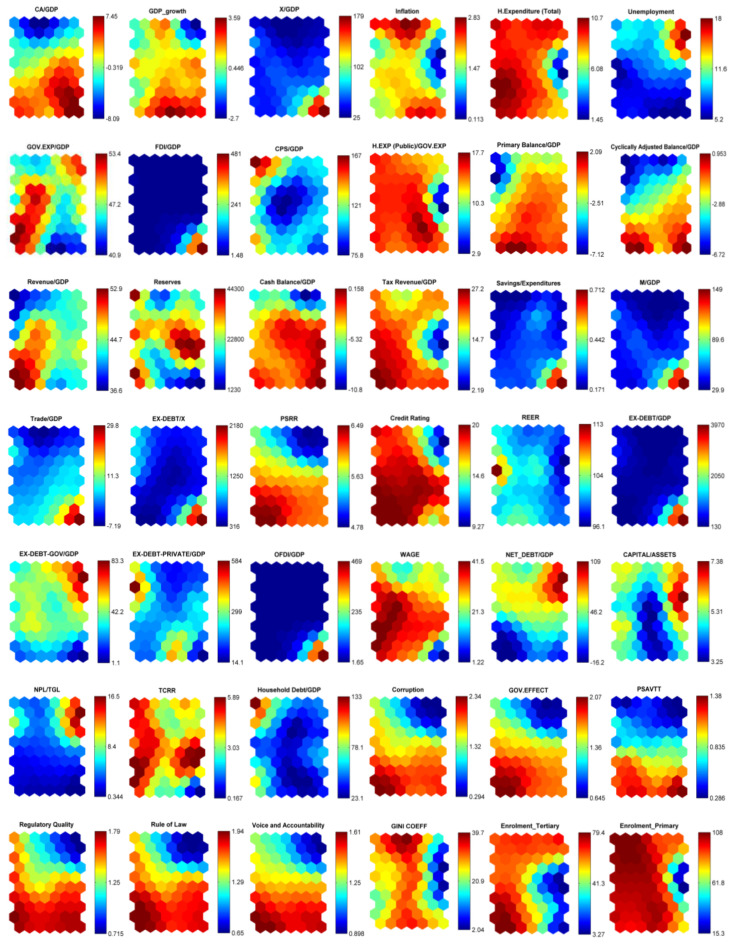

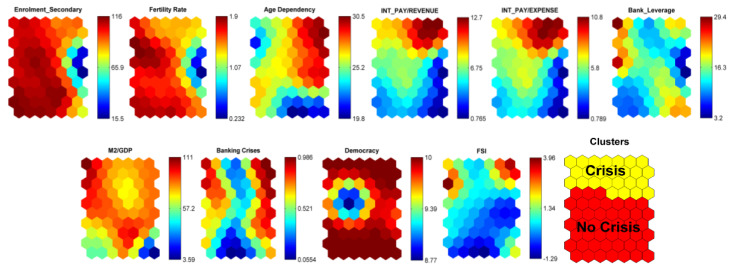
Net output of a SOM analysis: clusters based on unified distance matrix (U-matrix) and component matrixes.

We employ three different estimation techniques, namely SOM, logit, and Markov models. Unlike other econometric approaches, the SOM approach allows the researcher to work with large datasets and has the ability to visually monitor, via crisis maps created for each country for the period 2003–2015, the transition from no crisis (tranquil) to crisis states. Furthermore, through the SOM analysis, we are able to determine the variables’ order of importance in explaining the occurrence of the debt crises in the EU member countries. In other words, the SOM analysis serves as a filter to determine which indicators should be included in the logit and Markov model estimations. Figure 2 exhibits our results for a large number of 51 indicators using the SOM estimation method. As seen in Figure 2, each variable has its own component matrix with two-dimensional visuality. Temperature maps allow us to determine the value that each variable takes in crisis and noncrisis periods, obtained from the Davies–Bouldin index. The scale on the right-hand side of each graph (component matrix) increases the readability. To be more precise, each graph in Figure 2 represents the values for the different neurons of the respective variable using a color code ranging from dark blue (low values) to dark red (high values). Before interpreting the results of the SOM analysis, some aspects of the analysis require clarification. First, all countries (input) are placed in only one specific neuron (output) [66]. Since the time dimension of countries is also used in our analysis, the neuron in which the country is placed may change over the years. The analysis results show that countries with similar indicators are placed in the same or close neurons, while countries with different characteristics are placed in more distant neurons. When making interpretations, it is important to note that regardless of the variable analyzed, the location of the country is the same place, i.e., the same neuron. For example, the location of the neuron where Austria is located in 2015 is the same in the component matrix of all variables. Therefore, the value in the component matrix of that variable is interpreted according to the scale on the right side. Figure 2 shows the clusters of countries in the lower right corner. The weight vectors of the SOM neurons reveal the effect of each variable in determining the characteristics of the clusters [67]. The figure shows that there are two different clusters. The first cluster is the crisis cluster shown in yellow. The second cluster is the no crisis cluster shown in red.

Figure 2 shows that the likelihood of the debt crisis jumps with an increase in inflation, unemployment rate, budget deficit-to-GDP ratio, public and private external debt as the share of GDP, household debt, nonperforming loans, age dependency ratio, bank leverage, M2 over GDP, banking crisis index, and interest payments. Figure 2 also suggests that countries in debt crisis have low growth rates, low export-to-GDP ratio, low reserves, low shares of public revenues and taxes to GDP, and low credit ratings. Figure 2 shows the impact of governance indicators on the outbreak of the European debt crisis: estimates indicate that high income inequality, high corruption, low government effectiveness, low political stability risk-rating, low political stability (PSVATT), low regulatory quality, low rule of law, and low voice and accountability increase the crisis probability. FDI over GDP, the ratio of health expenditures to public expenditures, total health expenditures, savings/expenditures, the ratio of imports to GDP, the ratio of foreign trade balance to GDP, OFDI over GDP, capital over asset, and TCRR do not seem to have an effect on the occurrence of the European debt crisis. Finally, indicators related to education do not seem to have an impact on debt crises. It is worth highlighting that our SOM results are consistent with economic intuitions. The results of the SOM analysis are quite similar to the literature. Previous studies in the literature have found that increases in short-term debt, total external debt to GDP ratio, current account deficit to GDP ratio, inflation, level of reserves/GDP ratio, political problems, and trade openness increase the probability of debt crisis. They also find that decreases in foreign exchange reserves, real GDP growth, and primary and overall fiscal balance to GDP ratios are important determinants of debt crises [22,23,25,34,58,59,68,69].

In order to identify the unobserved relationships of the component matrixes, Table 4 exhibits the mean and standard deviation of each leading indicator in crisis and no crisis periods. Overall, results from the SOM analysis are consistent with the results presented in Table 4. For instance, the growth rates of countries in crisis zone tend to be low, leading to a decrease in these countries’ tax revenues and to a rise in both social transfers and unemployment benefits.

Table 5 and Table 6 present the ranking of 51 explanatory variables according to the six indexes selected in our study. In particular, results from the two overall indexes (Table 6) show that the ratio of nonperforming loans over total loans, primary balance over GDP, public sector borrowing requirement, corruption, cash balance over GDP, unemployment, voice and accountability, regulatory quality, rule of law, GDP growth, government effectiveness, and cyclically adjusted balance over GDP are the 10 most important indicators in explaining the outbreak of the European debt crisis. These 10 variables will be used in both logit and Markov estimation.

**Figure 3:**
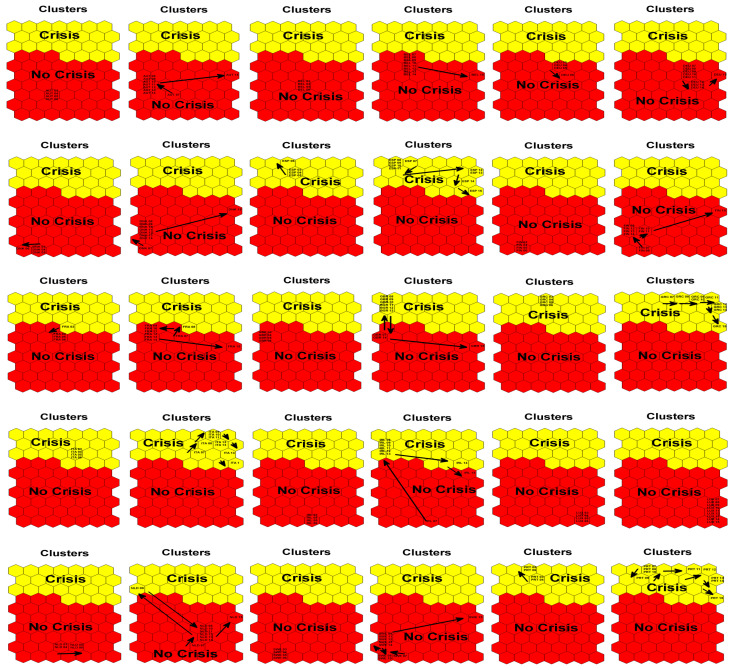
Self-organizing map results for EU-15 countries from 2003–2006 and 2007–2015.

In order to assess the extent to which the EU-15 countries have been affected by the crisis, we present the behavior of their economies on the maps from 2007 to 2015 (see Figure 3). Specifically, we sum up the above Figures and show the transition of EU-15 countries from no crisis to crisis states over time. Strikingly, we see that, when Europe was hit by the global financial crisis in 2007, Greece, Italy, Spain, and Portugal were already in the crisis zone.

The forecast performance results from the SOM estimates are presented in Table 7. The SOM model correctly predict 79.31% of crisis periods and 74% of the no crisis episodes in the EU-15 from 2003 to 2015. Importantly, the model forecasts 100% of crisis episodes for PIIGS countries.

After having obtained the 10 most significant variables that explain the debt crises from the SOM analysis, we estimate a logit model in which the dependent variable is the fiscal stress index reduced to a binary form. Note that the presence of the multicollinearity problem leads us to estimate each indicator separately.

Table 8 shows that all explanatory variables are statistically significant at 1% or 5%. According to the econometric results, increases in budget balance, PSRR, corruption, cash balance, voice and accountability, regulatory quality, GDP growth, rule of law, government effectiveness, and cyclically adjusted balance are associated with lower probabilities of crisis, while increases in NPL/TL and unemployment increase the likelihood of crisis. The results of the econometric analysis are quite similar to the literature. Manasse et al. [34] find that negative domestic developments (low real GDP growth and high inflation rates) and political factors increase the probability of debt crises. Hernandez de Cos et al. [23] find that fiscal balance over GDP and real GDP growth are important determinants of debt crisis. Bruns and Poghosyan [69] and Cerovic et al. [59] find that primary and overall fiscal balance to GDP ratios have a significant impact on debt crisis.

**Figure 4:**
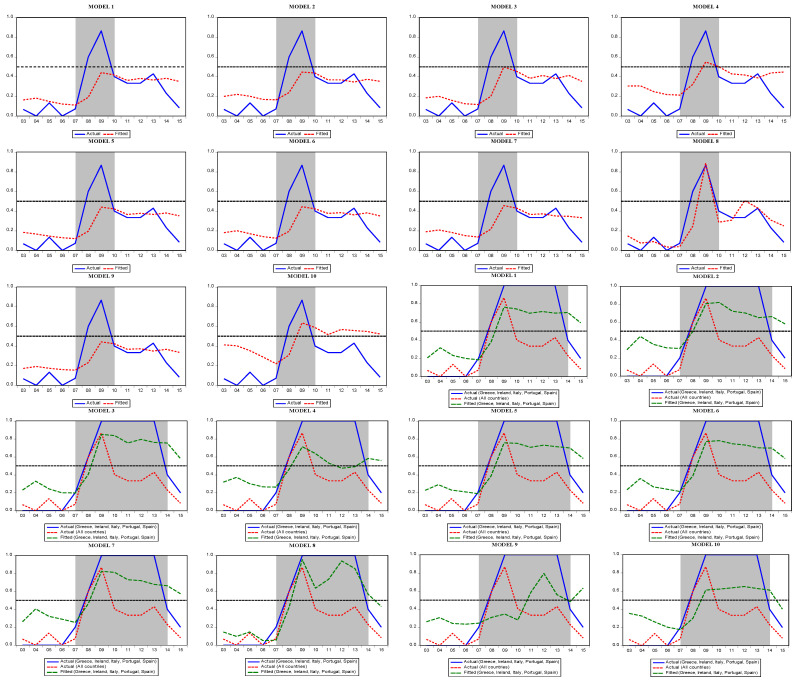
Predicted probability of crises in the logit models (EU-15 and PIIGS).

Figure 4 presents the actual and fitted values of the models estimated for the EU-15. We see that, except for Spain, Italy, Greece, Portugal, and Ireland, our studied countries experienced a crisis from 2007 to 2010. As expected, the crisis period was longer for PIIGS countries spanning the period 2007–2014. Table 9 presents the forecast performance matrices for the logit model. Accordingly, the success of the 10 models for predicting crises varies between 50% and 90% for different cut-off values.

In the panel Markov model, the dependent variable (the fiscal stress index) is a continuous variable. To avoid multicollinearity, each variable is estimated separately. Broadly speaking, the results obtained from the Markov approach (Table 10) are similar to those from the logit model. Table 10 suggests that NPL/TL, corruption, cash balance/GDP, voice and accountability, regulatory quality, rule of law, government effectiveness, and cyclically adjusted balance/GDP are statistically significant in only Regime 1, whereas primary balance/GDP, PSRR, unemployment, and GDP growth are statistically significant in Regimes 1 and 2. These results lead us to conclude that the ratios of NPL/TL and unemployment increase the likelihood of crisis, while increases in budget balance, PSRR, corruption, cash balance, voice and accountability, regulatory quality, GDP, and rule of law reduce the likelihood of crisis.

**Figure 5:**
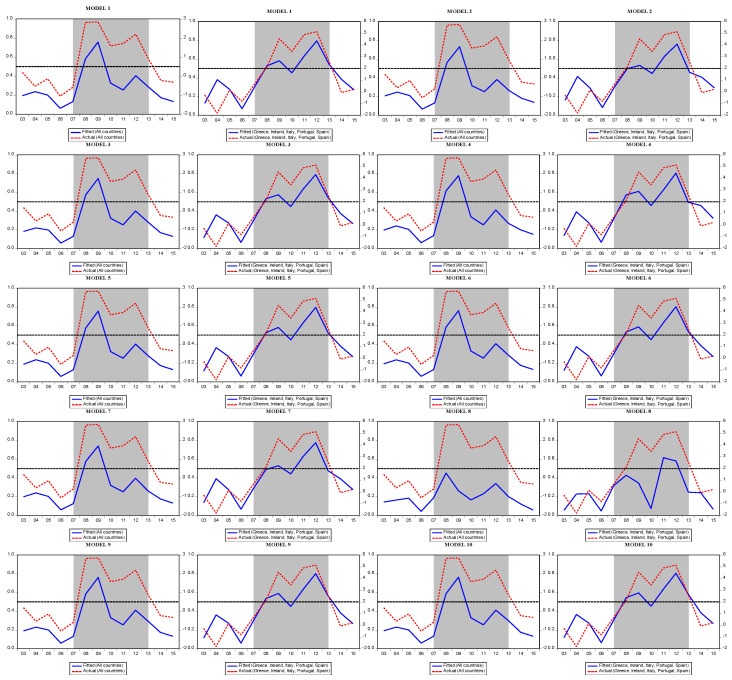
Predicted probability of crisis in the Markov regime switching models.

As in the logit model, the Markov model estimates also include the forecast performance of each model and the diagnostic test results. According to the results, there is no normality or autocorrelation problem in the estimated models. In addition, the linearity test shows that using the Markov regime switching model is more appropriate than the linear models. Crisis probabilities obtained from the Markov model are presented separately for the EU-15 and PIIGS. Unlike the logit model, Markov model forecasts show that the crisis started in late 2007 and lasted until 2013, both in PIIGS and the other 10 countries (Figure 5).

The forecast performance results obtained from the panel Markov model are given in Table 11 and Table 12. The models are able to predict, at 0.5 threshold level, all crisis episodes occurred in the EU-15 in the period of 2003–2015 and nearly 80% of no crisis periods. Model test results do not indicate any diagnostic problem and the linearity test results suggest that using nonlinear models such as the Markov and logit is appropriate to predict debt crisis (Table 13).

When we assess the forecast performance of different models, one should note that comparing the results obtained through the SOM with the logit and Markov forecasts can be misleading for two reasons. The first is that SOM uses 51 different leading indicators, while the logit and Markov model employ only 10. The second is that different thresholds cannot be used in the SOM approach. The forecast performance results from SOM show the model can predict crisis periods for the EU-15 more successfully than the no crisis periods. The forecast performance of the logit and Markov models differs according to the selected threshold value. But Markov estimates predict crisis periods more successfully than logit, while logit estimates predict no crisis periods more successfully than the Markov estimates. Markov models could predict approximately 100% of the crisis periods correctly, while the logit model predicted 100% of the no crisis periods (Selecting a lower threshold for both models improves the number of correctly predicted crisis periods but also causes non-crisis periods to be perceived as crises (Type II errors). Markov estimates can be said to have more Type II errors. In contrast, choosing a higher threshold value reduces the number of false alarms but at the expense of increasing the number of missed crises (Type I errors), particularly in logit models). 

## 4. Conclusions

This study aimed to empirically examine the European debt crisis. To do so, we first developed a fiscal stress index contrary for each EU-15 country within the period of 2003–2015, contrary to early empirical papers that tend to use event-based crisis indicators. The empirical results show that our fiscal stress index identifies more ‘debt crisis’ episodes and also indicates a longer crisis period, in particular for the so-called PIIGS, than previous empirical studies applied to debt crises (e.g., [21,22,23]).

As the results obtained from the SOM, Logit, and Markov models are very similar, we propose an overall interpretation. The similarity of the results obtained in all three models is an important indicator of consistency for our analysis. Empirical results obtained from three different models indicate that the debt crisis in the EU-15 is the consequence of the deterioration of both financial and macroeconomic variables such as nonperforming loans over total loans, GDP growth, unemployment rates, primary balance over GDP, and cyclically adjusted balance over GDP. Another interesting point in the estimation results is that despite the similar deterioration in macroeconomic variables, some European countries seem to have exited the crisis very quickly contrary to some countries like Portugal, Italy, Ireland, Spain, or Greece. When comparing these two sets of countries in detail, governance indicators are seen to have played an important role. This situation is observed from the fact that good governance indicators in the SOM, logit, and Markov results significantly reduced the possibility of debt crisis. Greece, Italy, Spain, Portugal, and Ireland, which were deeply affected by the crisis for a longer period, have all poor governance indicators. Therefore, the convergence of countries in terms of governance is very important in addition to economic convergence. Moreover, our logit and Markov models were quite successful in predicting the crisis episodes over the period of 2003–2015. To be more precise, nearly all crisis and no crisis periods in the EU-15 were correctly predicted by our models.

What are the policy implications of our findings? The first one is that constructing the continuous-time fiscal stress index which produces consistent and robust results in identifying fiscal pressure and/or crisis episodes may allow the authorities to take measures to prevent crises. The second one is that governance quality matters both in the outbreak and the length of debt crises. Hence, increasing governance quality could be a significant preventive response to future crises, and the EU may exert pressures on member countries to harmonize governance indicators. Moreover, when we analyze the movements of EU-15 countries over time in terms of macro, financial, and fiscal indicators, we find that there is no homogeneous structure. This can be easily observed from the figures obtained from the SOM analysis, which show the movements of these countries over time: Portugal, Italy, Ireland, Greece and Spain exhibit quite different economic indicators from other countries, not only during the financial and debt crises but also in the pre-crisis period of 2002–2006. Even in the pre-crisis period, these countries’ indicators were quite poor. Therefore, it is important that the countries within the European Union should be similar in terms of macro, financial and fiscal indicators.

Further studies can be carried out to include both a wider time period and a larger country set. In this way, more comprehensive results can be achieved for the constructed fiscal stress index and these results can be presented in a comparable way with previous studies. Furthermore, a very large set of indicators can be used to identify the factors that construct the fiscal stress index; it is thus possible to convert these indicators into the index by methods such as principal component analysis, factor analysis, unobserved components model and budget allocation process.

## Figures and Tables

**Table 1:** Optimal cut-off values for EU-15.

	Accuracy Measures			Sensitivity-Specificity Graphic			KLR	
Country	Cut-Off	Sensitivity	Specificity	Cut-Off	Sensitivity	Specificity	Cut-Off (*S*)	Cut-Off (*G*)
Austria	**0.410**	100.0	90.90	**0.410**	100.0	90.90	2.535	6.381
Belgium	2.376	50.0	90.90	**1.298**	100.0	90.90	3.343	6.381
Denmark	0.371	100.0	81.80	**2.254**	100.0	100.0	4.211	6.381
Finland	1.157	100.0	91.70	**3.433**	100.0	100.0	4.137	6.381
France	1.035	100.0	91.70	**1.788**	100.0	100.0	2.161	6.381
Germany	1.218	100.0	91.70	**3.516**	100.0	100.0	6.157	6.381
Greece	0.154	100.0	80.0	**0.752**	100.0	100.0	9.407	6.381
Ireland	−0.277	100.0	85.70	**0.435**	100.0	100.0	13.521	6.381
Italy	**0.229**	100.0	83.30	0.426	85.70	83.30	3.721	6.381
Luxembourg	3.855	100.0	91.70	**9.994**	100.0	100.0	10.985	6.381
Netherlands	1.058	100.0	90.90	**2.523**	100.0	100.0	3.972	6.381
Portugal	1.164	100.0	87.50	**1.729**	100.0	100.0	5.809	6.381
Spain	0.695	100.0	87.50	**1.998**	100.0	100.0	7.378	6.381
Sweden	−0.231	100.0	90.0	**0.275**	100.0	100.0	2.071	6.381
United Kingdom	0.991	100.0	90.0	**1.753**	100.0	100.0	3.748	6.381

**Note**: *S* and *G* indicate optimal threshold values for specific and all EU-15 countries, respectively.

**Table 2:** Debt crisis episodes from selected studies.

Country	Our Results: Crisis Dates	Hernandez de Cos et al. [23]: Crisis Dates	Baldacci et al. [21]: Start of Crisis	Berti et al. [22]
Austria	2009	No crisis	No crisis	No crisis
Belgium	2003, 2008–2009	No crisis	No crisis	No crisis
Denmark	2008–2009	n.a.	No crisis	No crisis
Finland	2009	No crisis	No crisis	No crisis
France	2009	No crisis	No crisis	No crisis
Germany	2005	No crisis	No crisis	No crisis
Greece	2008–2015	2008–2010	2008	n.a.
Ireland	2008–2013	2008–2010	2008	n.a.
Italy	2007–2014	2008–2010	2008	No crisis
Luxembourg	2008	n.a.	n.a.	No crisis
Netherlands	2008–2009	No crisis	No crisis	No crisis
Portugal	2009–2013	2008, 2010	2008, 2010	2009–2010
Spain	2009–2013	n.a.	2010	2009, 2012
Sweden	2009, 2013–2014	n.a.	No crisis	No crisis
United Kingdom	2008–2010	n.a.	No crisis	2009

**Note:** “n.a.” indicates that the country is not included in the study.

**Table 3:** Descriptive statistics of the dataset.

INDICATOR	ABBREVIATION	OBS	MIS.VAL.	MEAN	STD.DEV.	MIN	MAX
Current account of balance of payments (% of GDP)	CA/GDP ^1^	195	0(0%)	0.94	5.48	−14.43	11.93
GDP, real, annual growth	GDP growth ^1^	195	0(0%)	1.17	2.82	−9.17	8.40
Exports, goods & services (% of GDP)	X/GDP ^1^	195	0(0%)	54.52	39.96	18.54	213.85
Inflation, consumer prices index (annual %)	Inflation ^1^	195	0(0%)	1.79	1.36	−4.46	4.93
Health expenditure, total (% of GDP)	H. Expenditure (Total)/GDP ^1^	180	15(7.69%)	9.52	1.20	6.80	11.97
Unemployment rate (%)	Unemployment ^1^	195	0(0%)	8.46	4.65	2.33	27.51
Government expenditure as % of GDP	GOV.EXP/GDP ^1^	195	0(0%)	48.11	5.90	32.96	65.65
Foreign direct investment, inward, share of GDP	FDI/GDP ^1^	193	2(1.02%)	37.58	138.23	−6.75	1144.76
Domestic credit to private sector (% of GDP)	CPS/GDP ^1^	195	0(0%)	110.77	35.83	54.56	202.19
Health expenditure, public (% of government expenditure)	H.EXP (Public)/GOV.EXP ^1^	180	15(7.69%)	15.11	2.14	9.29	20.86
Primary net lending/borrowing (also referred as primary balance) (% of GDP)	Primary Balance/GDP ^2^	195	0(0%)	−0.94	3.71	−29.73	6.04
Cyclically adjusted balance (% of potential GDP)	Cyclically Adjusted Balance/GDP ^2^	195	0(0%)	−2.53	3.39	−18.61	4.01
Revenue (% of GDP)	Revenue/GDP ^2^	195	0(0%)	45.03	6.18	32.79	57.44
Reserves, foreign exchange, excluding gold, USD	Reserves ^1^	195	0(0%)	25,169.38	24,607.29	143.55	119,026
Cash surplus/deficit (% of GDP)	Cash Balance/GDP ^2^	179	16(8.20%)	−3.51	4.23	−32.37	4.11
Tax revenue (% of GDP)	Tax Revenue/GDP ^1^	179	16(8.20%)	22.22	5.85	0.31	35.08
Savings/Expenditures	Savings/Expenditures ^1^	194	1(0.51%)	0.28	0.14	0.08	0.85
Imports, goods & services (% of GDP)	M/GDP ^1^	195	0(0%)	50.45	31.81	22.92	177.65
Trade balance/GDP	Trade/GDP ^1^	195	0(0%)	4.08	9.10	−12.55	36.20
External debt, total, share of exports	EX-DEBT/X ^1^	190	5(2.56%)	673.46	498.41	258.78	2807.26
Political stability risk rating (7 = lowest risk)	PSRR ^3^	195	0(0%)	5.81	0.62	4.26	6.83
Credit rating, average	Credit Rating ^3^	195	0(0%)	17.88	4.15	0.00	20.00
Exchange rate, effective real	REER ^3^	195	0(0%)	101.52	5.46	88.99	127.40
External debt, total, share of GDP	EX-DEBT/GDP ^1^	190	5(2.56%)	511.16	983.55	82.98	5490.03
External debt government/GDP	EX-DEBT-GOV/GDP ^1^	179	16(8.20%)	41.96	25.61	1.65	152.47
External debt private/GDP	EX-DEBT-PRIVATE/GDP ^1^	177	18 (9.23%)	214.75	195.45	33.51	1067.07
Foreign direct investment, outward, share of GDP	OFDI/GDP ^1^	182	13(6.67%)	39.26	128.79	−3.95	833.68
Wages, hourly, USD	WAGE ^3^	182	13(6.67%)	32.44	10.10	8.19	51.67
Net debt (% of GDP)	NET_DEBT/GDP ^2^	164	21(10.77%)	42.88	47.10	−69.74	176.57
Bank capital to assets ratio (%)	CAPITAL/ASSETS ^1^	170	25(12.82%)	5.77	1.51	3.00	13.97
Bank nonperforming loans to total gross loans (%)	NPL/TGL ^1^	187	8(4.10%)	4.58	5.93	0.08	34.67
Trade credit risk rating (7 = lowest risk)	TCRR ^3^	152	23(11.79%)	5.32	1.99	0.00	7.00
Household Debt/GDP	Household Debt/GDP ^3^	125	70(35.90%)	84.16	36.15	46.78	217.51
Control of Corruption	Corruption ^1^	195	0(0%)	1.54	0.71	−0.25	2.55
Government Effectiveness	GOV.EFFECT ^1^	195	0(0%)	1.51	0.51	0.21	2.36
Political Stability and Absence of Violence/Terrorism	PSAVTT ^1^	195	0(0%)	0.81	0.46	−0.47	1.66
Regulatory Quality	Regulatory Quality ^1^	195	0(0%)	1.43	0.38	0.34	1.92
Rule of Law	Rule of Law ^1^	195	0(0%)	1.49	0.48	0.24	2.12
Voice and Accountability	Voice and Accountability ^1^	195	0(0%)	1.35	0.24	0.56	1.83
Gini coefficient	GINI COEFF ^4,5^	135	60(30.77%)	36.66	3.09	28.51	44.56
Gross enrolment ratio, tertiary, both sexes (%)	Enrolment Tertiary ^1^	156	39(20%)	67.44	16.33	10.33	110.26
Gross enrollment ratio, primary, both sexes (%)	Enrolment Primary ^1^	172	23(11.79%)	103.98	4.86	95.71	120.90
Gross enrolment ratio, secondary, both sexes (%)	Enrolment Secondary ^1^	172	23(11.79%)	110.46	13.14	91.39	164.81
Fertility rate, total (births per woman)	Fertility Rate ^1^	180	15(7.69%)	1.64	0.24	1.21	2.06
Age dependency ratio, old (% of working-age population)	Age Dependency ^1^	195	0(0%)	25.90	3.99	15.25	35.08
Interest payments (% of revenue)	INT_PAY/REVENUE ^1^	195	16(8.20%)	6.77	3.79	0.27	17.29
Interest payments (% of expense)	INT_PAY/EXPENSE ^1^	179	16(8.20%)	6.16	3.13	0.28	14.20
Banking sector leverage	Bank Leverage ^1^	180	15(7.69%)	16.03	9.52	3.89	51.56
M2/GDP	M2/GDP ^3^	182	13(6.67%)	81.31	22.09	41.62	133.32
Fiscal Stress Index	FSI ^6^	195	0(0%)	0.72	2.83	−9.78	15.99
Democracy	Democracy ^7^	195	0 (0%)	9.84	0.48	8.00	10.00
Index of Banking Crises (Laeven and Valencia, 2013)	Banking Crises	195	0(0%)	0.58	0.49	0.00	1.00

**Note:** Obs, Mis. Val, M, Min and Max denote observations, missing value, mean, minimum and maximum, respectively, while 1, 2, 3, 4, 5, 6, and 7 indicate World Bank, International Monetary Fund, Oxford Economics, World Income Inequality Database, and Standardized World Income Inequality.

**Table 4:** Self-organizing map-based cluster results.

VARIABLES	NO CRISIS (*M*)	CRISIS (*M*)	NO CRISIS (*SD*)	CRISIS (*SD*)
Frequency (%)	64.290	35.710	64.290	35.710
CA/GDP	3.566	−3.874	3.852	4.569
GDP Growth	1.754	0.066	2.527	3.017
X/GDP	64.514	35.858	43.348	23.399
Inflation	1.725	1.918	1.125	1.711
H. Expenditure (Total)/GDP	9.547	9.475	1.109	1.345
Unemployment	6.705	11.762	2.009	6.175
GOV.EXP/GDP	48.450	47.522	6.285	5.095
FDI/GDP	55.846	3.998	169.145	6.140
CPS/GDP	104.953	121.710	34.557	35.854
H.EXP (Public)/GOV.EXP	15.651	14.096	2.0931	1.941
Primary Balance/GDP	0.115	−2.949	2.264	4.919
Cyclically Adjusted Balance/GDP	−1.184	−5.046	2.289	3.683
Revenue/GDP	47.309	40.774	5.796	4.403
Reserves	27,640.150	20,564.050	24,962.110	23,407.870
Cash Balance/GDP	−1.956	−6.317	2.271	5.378
Tax Revenue/GDP	23.469	19.660	5.451	6.406
Savings/Expenditures	0.308	0.218	0.147	0.117
M/GDP	57.648	36.955	35.300	17.497
Trade/GDP	6.866	−1.097	8.773	7.232
EX-DEBT/X	674.356	670.413	588.113	269.289
PSRR	6.154	5.171	0.303	0.530
Credit Rating	19.254	15.310	3.091	4.645
REER	102.214	100.212	6.097	3.733
EX-DEBT/GDP	644.485	266.116	1188.315	257.153
EX-DEBT-GOV/GDP	34.552	54.269	19.030	30.138
EX-DEBT-PRIVATE/GDP	216.908	211.008	149.895	254.582
OFDI/GDP	60.280	4.005	159.224	6.483
WAGE	37.241	24.375	6.667	9.795
NET_DEBT/GDP	23.572	77.249	40.604	37.454
CAPITAL/ASSETS	5.532	6.149	1.567	1.330
NPL/TGL	2.177	8.817	1.874	7.942
TCRR	5.945	4.367	1.708	2.025
Household Debt/GDP	71.747	108.856	27.480	39.372
Corruption	1.922	0.817	0.367	0.631
GOV.EFFECT	1.790	0.981	0.246	0.445
PSAVTT	1.011	0.421	0.333	0.423
Regulatory Quality	1.615	1.082	0.218	0.378
Rule of Law	1.752	1.014	0.213	0.476
Voice and Accountability	1.483	1.109	0.138	0.205
GINI COEFF	35.677	38.523	2.752	2.888
Enrollment Tertiary	66.145	69.360	17.513	13.812
Enrollment Primary	103.028	105.829	4.228	5.458
Enrollment Secondary	111.447	108.453	14.072	11.030
Fertility Rate	1.714	1.508	0.197	0.266
Age Dependency	25.413	26.806	3.725	4.334
INT_PAY/REVENUE	4.695	10.524	2.241	3.072
INT_PAY/EXPENSE	4.571	9.026	2.100	2.607
Bank Leverage	15.474	17.058	8.312	11.362
M2/GDP	77.711	87.144	23.254	18.801
FSI	0.038	1.981	2.394	3.154
Democracy	9.772	9.971	0.566	0.170
Banking Crises	0.520	0.691	0.502	0.465

**Table 5:** List of significant variables ranked based on four indexes (SI—structuring index, RI—relative importance, CD—cluster description, and SRC—Spearman’s rank correlation) in a SOM.

Rank	SI	Values	RI	Values	CD	Values	SRC	Values
1	GOV.EFFECT	1328.206	Primary Balance/GDP	2.487	NPL/TGL	4.238	GDP growth	−0.639 ***
2	PSRR	1320.574	EX-DEBT-GOV/GDP	2.370	Unemployment	3.074	Primary Balance/GDP	−0.527 ***
3	Voice and Accountability	1313.764	PSRR	2.347	Cash Balance/GDP	2.368	Cash Balance/GDP	−0.428 ***
4	Rule of Law	1313.293	Corruption	2.329	Rule of Law	2.235	Cyclically Adjusted Balance/GDP	−0.398 ***
5	Corruption	1310.869	NET_DEBT/GDP	2.283	Primary Balance/GDP	2.173	NPL/TGL	0.386 ***
6	Regulatory Quality	1282.465	Unemployment	2.231	GOV.EFFECT	1.805	Banking Crises	0.373 ***
7	CA/GDP	1241.308	Regulatory Quality	2.194	PSRR	1.746	EX-DEBT/X	0.341 ***
8	INT_PAY/REVENUE	1215.579	M2/GDP	2.194	Regulatory Quality	1.736	CA/GDP	−0.324 ***
9	PSAVTT	1192.348	CAPITAL/ASSET	2.182	Corruption	1.722	Bank Leverage	0.323 ***
10	INT_PAY/EXPENSE	1176.359	INT_PAY/EXPENSE	2.181	EX-DEBT-PRIVATE/GDP	1.698	GOV.EFFECT	−0.313 ***
11	NET_DEBT/GDP	1097.219	Cash Balance/GDP	2.168	Cyclically Adjusted Balance/GDP	1.609	PSRR	−0.306 ***
12	Trade/GDP	1048.616	GINI COEFF	2.136	EX-DEBT-GOV/GDP	1.584	Voice and Accountability	−0.302 ***
13	Age Dependency	1023.416	Reserves	2.132	Inflation	1.522	NET_DEBT/GDP	0.302 ***
14	Cyclically Adjusted Balance/GDP	992.023	Enrollment Tertiary	2.131	Credit Rating	1.503	Savings/Expenditures	−0.295 ***
15	WAGE	983.387	CPS/GDP	2.127	Voice and Accountability	1.491	EX-DEBT-GOV/GDP	0.280 ***
16	Revenue/GDP	927.769	Bank Leverage	2.120	WAGE	1.469	INT_PAY/REVENUE	0.269 ***
17	Enrollment Tertiary	927.575	Banking Crises	2.114	Household Debt/GDP	1.433	Rule of Law	−0.268 ***
18	NPL/TGL	926.662	Voice and Accountability	2.097	INT_PAY/REVENUE	1.371	TCRR	−0.267 ***
19	Unemployment	908.386	GDP growth	2.085	Bank Leverage	1.367	Trade/GDP	−0.262 ***
20	X/GDP	907.778	EX-DEBT/GDP	2.078	Fertility Rate	1.350	OFDI/GDP	−0.260 ***
21	Tax Revenue/GDP	904.0651	Trade/GDP	2.057	FSI	1.317	Corruption	−0.255 ***
22	Fertility Rate	892.051	Enrollment Secondary	2.046	Enrollment Primary	1.291	Credit Rating	−0.255 ***
23	M/GDP	868.569	NPL/TGL	2.016	PSAVTT	1.270	M2/GDP	0.249 ***
24	Cash Balance/GDP	865.003	FDI/GDP	1.98	INT_PAY/EXPENSE	1.242	PSAVTT	−0.247 ***
25	Democracy	853.076	TCRR	1.977	H. Expenditure (Total)/GDP	1.213	Household Debt/GDP	0.231 ***
26	EX-DEBT-GOV/GDP	852.431	H.EXP (Public)/GOV.EXP	1.917	GDP growth	1.194	Unemployment	0.229 ***
27	GOV.EXP/GDP	833.429	H. Expenditure (Total)/GDP	1.908	CA/GDP	1.186	GOV.EXP/GDP	0.223 ***
28	EX-DEBT/X	822.099	Inflation	1.903	TCRR	1.185	Regulatory Quality	−0.211 ***
29	M2/GDP	813.392	OFDI/GDP	1.881	Tax Revenue/GDP	1.175	X/GDP	−0.192 ***
30	Credit Rating	810.121	Enrollment Primary	1.873	Age Dependency	1.163	Enrolment Primary	0.186 **
31	Banking Crises	787.017	Age Dependency	1.854	GINI COEFF	1.045	M/GDP	−0.173 **
32	H. Expenditure (Total)/GDP	774.519	Tax Revenue/GDP	1.849	CPS/GDP	1.038	INT_PAY/EXPENSE	0.171 **
33	Savings/Expenditures	761.388	Cyclically Adjusted Balance/GDP	1.847	Reserves	0.938	GINI COEFF	0.166 *
34	Bank Leverage	756.183	Revenue/GDP	1.835	Banking Crises	0.928	EX-DEBT/GDP	0.153 **
35	EX-DEBT-PRIVATE/GDP	756.152	REER	1.829	H.EXP (Public)/GOV.EXP	0.927	Revenue/GDP	−0.152 **
36	CPS/GDP	738.579	CA/GDP	1.801	NET_DEBT/GDP	0.922	H.EXP (Public)/GOV.EXP	−0.140 *
37	GINI COEFF	712.864	Household Debt/GDP	1.791	CAPITAL/ASSETS	0.849	Age Dependency	0.105
38	Reserves	707.545	Credit Rating	1.759	Trade/GDP	0.824	Tax Revenue/GDP	−0.103
39	H.EXP (Public)/GOV.EXP	700.329	Fertility Rate	1.755	GOV.EXP/GDP	0.811	FDI/GDP	−0.101
40	Enrollment Secondary	683.320	GOV.EXP/GDP	1.745	M2/GDP	0.809	CPS/GDP	0.090
41	Primary Balance/GDP	682.901	Rule of Law	1.735	Savings/Expenditures	0.793	H. Expenditure (Total)/GDP	0.087
42	Enrollment Primary	667.923	X/GDP	1.730	Enrollment Tertiary	0.789	Reserves	−0.071
43	EX-DEBT/GDP	647.172	PSAVTT	1.691	Enrollment Secondary	0.784	Enrollment Secondary	0.061
44	GDP growth	642.788	GOV.EFFECT	1.672	Revenue/GDP	0.760	CAPITAL/ASSET	−0.059
45	CAPITAL/ASSETS	638.174	EX-DEBT/X	1.551	REER	0.612	WAGE	−0.058
46	FSI	615.079	FSI	1.541	X/GDP	0.540	Fertility Rate	−0.058
47	TCRR	611.866	INT_PAY/REVENUE	1.497	M/GDP	0.496	EX-DEBT-PRIVATE/GDP	0.034
48	Inflation	608.657	Savings/Expenditures	1.488	EX-DEBT/X	0.458	REER	0.024
49	Household Debt/GDP	567.960	M/GDP	1.477	Democracy	0.301	Democracy	−0.026
50	REER	564.612	Democracy	1.473	EX-DEBT/GDP	0.216	Enrollment Tertiary	−0.020
51	OFDI/GDP	531.554	WAGE	1.341	OFDI/GDP	0.041	Inflation	0.012
52	FDI/GDP	484.981	EX-DEBT-PRIVATE/GDP	1.193	FDI/GDP	0.036		

**Note:** ***, ** and * represent statistical significance at the 1%, 5% and 10% level, respectively.

**Table 6:** List of significant variables ranked based on SRC (crisis and non-crisis periods)—Spearman’s rank correlation and overall indexes) in a SOM.

Rank	SRC (Crisis)	Values	SRC (No Crisis)	Values	Overall Index (1)	Values	Overall Index (2)	Values
1	GDP growth	−0.752 ***	Primary Balance/GDP	−0.543 ***	NPL/TGL	14.112	NPL/TGL	5.992
2	Banking Crises	0.547 ***	GDP growth	−0.510 ***	Primary Balance/GDP	12.135	Primary Balance/GDP	4.780
3	Household Debt/GDP	0.516 ***	Cyclically Adjusted Balance/GDP	−0.315 **	Cash Balance/GDP	11.617	PSRR	4.769
4	EXDEBT/GDP	0.512 ***	Cash Balance/GDP	−0.284 ***	GDP growth	11.148	Corruption	4.257
5	EXDEBT/X	0.490 ***	Bank Leverage	0.263 ***	Unemployment	11.059	Cash Balance/GDP	3.969
6	Primary Balance/GDP	−0.476 ***	Trade/GDP	−0.259 ***	PSRR	10.725	Unemployment	3.902
7	GOV.EXP/GDP	0.471 ***	WAGE	0.251 ***	Rule of Law	10.508	Voice and Accountability	3.472
8	Bank Leverage	0.459 ***	Banking Crises	0.251 ***	GOV.EFFECT	10.221	Regulatory Quality	3.357
9	EXDEBTPRIVATE/GDP	0.401 ***	EXDEBT/X	0.239 ***	Corruption	10.185	Rule of Law	2.978
10	M2/GDP	0.393 ***	GOV.EXP/GDP	0.238 ***	Cyclically Adjusted Balance/GDP	10.128	GDP growth	2.645
11	Cash Balance/GDP	−0.333 ***	GINI COEFF	0.215 **	Voice and Accountability	10.029	GOV.EFFECT	2.548
12	NPL/TGL	0.320 ***	Savings/Expenditures	−0.194 **	Regulatory Quality	9.609	EX-DEBT-GOV/GDP	2.453
13	Savings/Expenditures	−0.314 ***	CA/GDP	−0.192 **	CA/GDP	9.302	NET_DEBT/GDP	2.420
14	Enrollment Secondary	0.305 **	NPL/TGL	0.192 **	EX-DEBT-GOV/GDP	9.234	Cyclically Adjusted Balance/GDP	2.094
15	X/GDP	0.302 **	EXDEBTGOV/GDP	0.172 *	NET_DEBT/GDP	8.860	INT_PAY/EXPENSE	1.880
16	Cyclically Adjusted Balance/GDP	−0.264 *	CAPITAL/ASSETS	−0.161 *	Bank Leverage	8.828	CA/GDP	1.855
17	GINI COEFF	−0.262 **	FSI	1	INT_PAY/REVENUE	8.728	Bank Leverage	1.174
18	Unemployment	0.260 **	Democracy	−0.156	Banking Crises	8.665	Banking Crises	1.038
19	Credit Rating	−0.240 **	X/GDP	−0.145	PSAVTT	8.515	Trade/GDP	0.985
20	EXDEBTGOV/GDP	0.239 **	OFDI/GDP	−0.145	INT_PAY/EXPENSE	8.236	PSAVTT	0.811
21	Fertility Rate	0.226 *	EXDEBT/GDP	0.139	Credit Rating	8.178	INT_PAY/REVENUE	0.526
22	CAPITAL/ASSETS	−0.220 *	M2/GDP	0.117	Trade/GDP	8.012	M2/GDP	0.358
23	M/GDP	0.211 *	TCRR	−0.100	TCRR	7.578	Credit Rating	−0.182
24	INT_PAY/EXPENSE	−0.210 *	Tax Revenue/GDP	−0.099	M2/GDP	7.492	Age Dependency	−0.517
25	FSI	1	H. Expenditure (Total)/GDP	0.097	Household Debt/GDP	7.352	GINI COEFF	−0.551
26	Inflation	−0.188	M/GDP	−0.094	EX-DEBT/X	7.161	TCRR	−0.606
27	Trade/GDP	0.173	Enrollment Primary	−0.093	Savings/Expenditures	7.065	Tax Revenue/GDP	−1.037
28	WAGE	0.171	PSAVTT	−0.085	Enrollment Primary	7.025	CPS/GDP	−1.041
29	OFDI/GDP	−0.168	Enrollment Tertiary	0.085	GOV.EXP/GDP	6.854	Enrollment Tertiary	−1.087
30	H.EXP (Public)/GOV.EXP	−0.162	Rule of Law	−0.068	Age Dependency	6.852	Enrollment Primary	−1.182
31	H. Expenditure (Total)/GDP	0.157	Unemployment	0.065	GINI COEFF	6.824	Revenue/GDP	−1.206
32	Reserves	−0.152	Reserves	0.062	Tax Revenue/GDP	6.585	GOV.EXP/GDP	−1.332
33	Tax Revenue/GDP	0.142	NET_DEBT/GDP	0.062	Revenue/GDP	6.427	Household Debt/GDP	−1.367
34	REER	0.140	INT_PAY/REVENUE	0.057	Fertility Rate	6.327	Reserves	−1.433
35	CPS/GDP	0.135	FDI/GDP	−0.057	X/GDP	6.301	H. Expenditure (Total)/GDP	−1.441
36	NET_DEBT/GDP	0.119	Voice and Accountability	−0.056	WAGE	6.297	Fertility Rate	−1.506
37	Enrollment Primary	0.117	REER	0.053	H. Expenditure (Total)/GDP	6.274	X/GDP	−1.676
38	Regulatory Quality	−0.109	Age Dependency	0.052	CPS/GDP	6.170	EX-DEBT/X	−1.694
39	Enrollment Tertiary	0.084	Household Debt/GDP	−0.051	H.EXP (Public)/GOV.EXP	6.159	H.EXP (Public)/GOV.EXP	−1.734
40	Rule of Law	0.078	GOV.EFFECT	−0.049	EX-DEBT-PRIVATE/GDP	5.787	CAPITAL/ASSET	−1.762
41	Voice and Accountability	−0.07	Enrollment Secondary	0.048	Reserves	5.780	Savings/Expenditures	−2.043
42	FDI/GDP	0.052	EXDEBTPRIVATE/GDP	0.048	M/GDP	5.721	Enrollment Secondary	−2.128
43	CA/GDP	0.049	H.EXP (Public)/GOV.EXP	0.041	Inflation	5.701	Inflation	−2.279
44	INT_PAY/REVENUE	0.032	CPS/GDP	−0.040	Enrollment Tertiary	5.568	EX-DEBT/GDP	−2.285
45	TCRR	−0.031	PSRR	−0.036	OFDI/GDP	5.473	WAGE	−2.418
46	Revenue/GDP	−0.027	Inflation	0.034	CAPITAL/ASSET	5.431	OFDI/GDP	−2.931
47	Age Dependency	0.021	Regulatory Quality	0.033	Enrollment Secondary	5.312	M/GDP	−2.937
48	Corruption	0.013	Fertility Rate	−0.027	EX-DEBT/GDP	5.222	EX-DEBT-PRIVATE/GDP	−3.759
49	GOV.EFFECT	−0.012	Revenue/GDP	0.024	FSI	4.927	FSI	−3.906
50	PSRR	0.005	Corruption	−0.016	REER	4.232	REER	−3.908
51	PSAVTT	0.005	INT_PAY/EXPENSE	−0.012	Democracy	4.046	FDI/GDP	−3.950
52	Democracy	0.004	Credit Rating	0.010	FDI/GDP	4.017	Democracy	−4.365

**Note:** ***, ** and * represent statistical significance at the 1%, 5% and 10% level, respectively.

**Table 7:** Forecast performance of SOM.

Criteria	Model (EU-15)	Model (PIIGS)
% and number of correctly predicted non-crises	79.31% (115/145)	18.18% (6/33)
% and number of correctly predicted crises	74.00% (37/50)	100% (32/32)

**Table 8:** Logit estimation results.

Dependent Variable: FSI										
Variables	Model 1	Model 2	Model 3	Model 4	Model 5	Model 6	Model 7	Model 8	Model 9	Model 10
NPL/TGL	0.150 *** (0.038)	0.063 ** (0.028)	0.167 *** (0.052)	0.164 *** (0.052)	0.131 *** (0.036)	0.108 *** (0.033)	0.096 *** (0.031)	0.168 *** (0.050)	0.093 *** (0.030)	0.175 *** (0.042)
Primary Balance/GDP	−0.280 *** (0.071)	−0.251 *** (0.1348)	−0.153 * (0.081)	−0.277 *** (0.073)	−0.265 *** (0.069)	−0.266 *** (0.069)	−0.285 *** (0.071)	−0.162 ** (0.083)	−0.259 *** (0.070)	−0.249 ** (0.103)
PSRR	−0.375 *** (0.052)									
Corruption		−1.094 *** (0.153)								
Cash Balance/GDP			−0.173 ** (0.076)							
Unemployment				0.024 *** (0.004)						
Voice and Accountability					−1.537 *** (0.211)					
Regulatory Quality						−1.361 *** (0.187)				
Rule of Law							−1.296 *** (0.178)			
GDP growth								−0.612 *** (0.123)		
GOV.EFFECT									−1.260 *** (0.174)	
Cyclically Adjusted Balance/GDP										−0.165 *** (0.039)
CONSTANT	1.323 (2.335)	−1.541 ** (0.697)	−2.605 *** (0.374)	−2.390 *** (0.428)	−0.047 (1.613)	−1.240 (0.999)	−0.913 (0.814)	−1.713 *** (0.366)	−0.985 (0.829)	−2.333 *** (0.351)
Observations	195	195	195	195	195	195	195	195	195	195
Pseudo R^2^	0.27	0.26	0.30	0.26	0.27	0.26	0.27	0.45	0.27	0.26
LR Stat	59.4 ***	58.4 ***	63.7 ***	57.4 ***	59.0 ***	58.2 ***	60.0 ***	99.7 ***	59.6 ***	57.4 ***
Akaike Info	0.91	0.91	0.89	0.92	0.91	0.91	0.90	0.69	0.91	0.92

**Note:** ***, ** and * represent statistical significance at the 1%, 5% and 10% level, respectively. The values in parentheses are standard deviations.

**Table 9:** Forecast performance of logit models.

Cut-Off Level	Model 1	Model 2	Model 3	Model 4	Model 5	Model 6	Model 7	Model 8	Model 9	Model 10
C = 0.5										
% and number of correctly predicted non-crises	95.10% (136/143)	93.71% (134/143)	95.10% (136/143)	83.22% (119/143)	95.10% (136/143)	95.10% (136/143)	95.10% (136/143)	97.20% (139/143)	93.71% (134/143)	66.43% (95/143)
% and number of correctly predicted crises	50% (26/52)	55.77% (29/52)	57.69% (30/52)	51.92% (27/52)	50% (26/52)	55.77% (29/52)	55.77% (29/52)	67.31% (35/52)	55.77% (29/52)	53.85% (28/52)
C = 0.25										
% and number of correctly predicted non-crises	76.22% (109/143)	74.83% (107/143)	85.52% (118/143)	40.56% (58/143)	75.52% (108/143)	72.03% (103/143)	76.22% (109/143)	85.31% (122/143)	75.52% (108/143)	21.68% (31/143)
% and number of correctly predicted crises	69.23% (36/52)	73.08% (38/52)	75% (39/52)	76.92% (40/52)	69.23% (36/52)	69.23% (36/52)	73.08% (38/52)	78.85% (41/52)	73.08% (38/52)	88.46% (46/52)
C = 0.2										
% and number of correctly predicted non-crises	68.53% (98/143)	66.43% (95/143)	72.72% (104/143)	34.27% (49/143)	67.83% (97/143)	69.93% (100/143)	65.73% (94/143)	78.32% (112/143)	69.93% (100/143)	13.94% (20/143)
% and number of correctly predicted crises	76.92% (40/52)	75% (39/52)	76.92% (40/52)	88.46% (46/52)	75% (39/52)	73.08% (38/52)	75% (39/52)	78.85% (41/52)	76.92% (40/52)	90.38% (47/52)

**Table 10:** Markov Estimation Results.

Variables	Model 1	Model 2	Model 3	Model 4	Model 5	Model 6	Model 7	Model 8	Model 9	Model 10
NPL/TGL (Regime 1)	0.0852 *** (4.6928)	0.0860 *** (4.6168)	0.0840 *** (5.6005)	0.0691 ** (2.5123)	0.0799 *** (4.3170)	0.0824 *** (4.4995)	0.0740 *** (4.4310)	0.0429 *** (2.9431)	0.0850 *** (4.6662)	0.0840 *** (5.6520)
NPL/TGL (Regime 2)	0.0020 (0.0193)	0.0081 (0.0748)	0.0223 (0.2283)	0.0170 (0.1403)	0.0064 (0.0550)	0.0120 (0.1178)	0.0057 (0.0551)	0.0364 (0.3093)	0.0005 (0.0049)	0.0398 (0.4684)
Primary Balance/GDP (Regime 1)	−0.3012 *** (−4.2105)	−0.0316 *** (−9.8841)	−0.2882 *** (−7.0861)	−0.3030 *** (−10.6341)	−0.2977 *** (−9.6040)	−0.2997 *** (−10.0140)	−0.2967 *** (−9.7860)	−0.2192 *** (−11.2549)	−0.3010 *** (−9.6911)	−0.3076 *** (−7.4115)
Primary Balance/GDP (Regime 2)	−0.2277 ** (−2.0433)	−0.2347 ** (−2.0862)	−0.2100 * (−1.7335)	−0.2210 * (−1.9493)	−0.2290 ** (−2.0338)	−0.2362 ** (−2.0885)	−0.2399 ** (−2.0749)	−0.1864 (−0.9240)	−0.2273 ** (−2.0389)	−0.1602 (−1.0865)
PSRR (Regime 1)	−0.5258 ** (−2.3573)									
PSRR (Regime 2)	−1.6070 * (−1.6448)									
Corruption (Regime 1)		−0.5181 *** (−2.8380)								
Corruption (Regime 2)		−0.8653 (−0.9782)								
Cash Balance/GDP (Regime 1)			−0.1913 *** (−6.4542)							
Cash Balance/GDP (Regime 2)			−0.0698 (−0.75690)							
Unemployment (Regime 1)				0.0893 ** (2.1616)						
Unemployment (Regime 2)				0.1897 * (1.8022)						
Voice and Accountability (Regime 1)					−1.8370 *** (−3.6224)					
Voice and Accountability (Regime 2)					−3.2583 (−1.2709)					
Regulatory Quality (Regime 1)						−1.0375 *** (−2.8681)				
Regulatory Quality (Regime 2)						−1.5038 (−0.9755)				
Rule of Law (Regime 1)							−0.8168 *** (−3.0846)			
Rule of Law (Regime 2)							−1.3201 (−0.9453)			
GDP growth (Regime 1)								−0.4262 *** (−11.7979)		
GDP growth (Regime 2)								−0.5844 *** (−3.4714)		
GOV.EFFECT (Regime 1)									−0.6890 ** (−2.4132)	
GOV.EFFECT (Regime 2)									−1.9062 (−1.5911)	
Cyclically Adjusted Balance/GDP (Regime 1)										−0.2166 *** (−6.4593)
Cyclically Adjusted Balance/GDP (Regime 2)										−0.2517 (−1.5493)
CONSTANT (Regime 1)	3.0353 ** (2.3498)	0.7753 ** (2.4738)	−0.6157 *** (−4.8374)	−0.7358 ** (−2.2208)	2.4941 *** (3.5863)	1.4636 *** (2.7435)	1.2027 *** (2.8605)	0.7648 *** (7.2357)	1.0208 ** (2.2128)	−0.5427 *** (−4.6762)
CONSTANT (Regime 2)	11.6515 ** (2.1514)	3.9409 *** (2.9538)	2.3620 ** (2.5289)	0.7188 (0.5726)	6.9388 ** (2.1365)	4.9064 ** (2.3032)	4.6355 ** (2.3107)	2.4270 *** (4.1550)	5.2348 *** (3.0322)	1.7041 ** (2.0224)

**Note:** ***, ** and * represent statistical significance at the 1%, 5% and 10% levels, respectively. The values in parentheses are *t*-values.

**Table 11:** Forecast performance of PMRSM (EU-15).

Cut-Off Level	Model 1	Model 2	Model 3	Model 4	Model 5	Model 6	Model 7	Model 8	Model 9	Model 10
C = 0.5										
% and number of correctly predicted non-crises	100%	100%	100%	100%	100%	100%	100%	100%	100%	100%
% and number of correctly predicted crises	100%	100%	100%	100%	100%	100%	100%	0%	100%	100%
C = 0.25										
% and number of correctly predicted non-crises	72.72%	54.54%	63.63%	63.63%	63.63%	63.63%	63.63%	90.90%	63.63%	72.72%
% and number of correctly predicted crises	100%	100%	100%	100%	100%	100%	100%	100%	100%	100%
C = 0.2										
% and number of correctly predicted non-crises	54.54%	45.45%	45.45%	27.27%	45.45%	45.45%	36.36%	81.81%	45.45%	54.54%
% and number of correctly predicted crises	100%	100%	100%	100%	100%	100%	100%	100%	100%	100%

**Table 12:** Forecast performance of PMRSM (Greece, Ireland, Italy, Portugal, and Spain).

Cut-Off Level	Model 1	Model 2	Model 3	Model 4	Model 5	Model 6	Model 7	Model 8	Model 9	Model 10
C = 0.5										
% and number of correctly predicted non-crises	100%	100%	100%	100%	100%	100%	100%	100%	100%	100%
% and number of correctly predicted crises	66.66%	66.66%	83.33%	66.66%	83.33%	83.33%	50.00%	33.33%	83.33%	83.33%
C = 0.25										
% and number of correctly predicted non-crises	42.86%	28.57%	28.57%	28.57%	28.57%	28.57%	28.57%	85.71%	28.57%	28.57%
% and number of correctly predicted crises	100%	100%	100%	100%	100%	100%	100%	83.33%	100%	100%
C = 0.2										
% and number of correctly predicted non-crises	42.86%	28.57%	28.57%	28.57%	28.57%	28.57%	28.57%	57.14%	28.57%	28.57%
% and number of correctly predicted crises	100%	100%	100%	100%	100%	100%	100%	83.33%	100%	100%

**Table 13:** Test statistics for the Markov regime switching models.

Models	Model 1	Model 2	Model 3	Model 4	Model 5	Model 6	Model 7	Model 8	Model 9	Model 10
Sigma 0	−0.3609	−0.3618	−0.3417	−0.3967	−0.3563	−0.3652	−0.3619	−0.3880	−0.3605	−0.3658
Sigma 1	1.4222	1.4276	1.6380	1.4162	1.4281	1.4235	1.4327	1.5241	1.4219	1.4227
P_00_	0. 7944	0. 7942	0.8001	0.7859	0.7953	0.7937	0.7959	0.8327	0.7944	0.7940
P_11_	0.4819	0.4800	0.4790	0.4826	0.4746	0.4800	0.4705	0.3092	0.4816	0.4843
Log-Likelihood	−365.36	−365.51	−365.54	−364.98	−365.04	−365.17	−363.92	−327.11	−365.35	−365.50
Linearity Test $\chi^{2}$ (7)	168.13 ***	168.60 ***	168.32 ***	168.17 ***	168.84 ***	168.79 ***	170.34 ***	204.02 ***	168.15 ***	168.59 ***
Portmanteau Serial correlation $\chi^{2}$ (6)	19.66 [0.10]	21.30 [0.07]	19.45 [0.11]	21.41 [0.07]	21.26 [0.07]	20.68 [0.08]	24.23 [0.03]	9.46 [0.73]	19.33 [0.11]	19.63 [0.10]
Doornik and Hansen Normality $\chi^{2}$ (2)	4.27 [0.12]	4.51 [0.10]	5.47 [0.06]	7.16 [0.03]	3.89 [0.14]	4.97 [0.08]	3.88 [0.14]	3.24 [0.20]	4.33 [0.11]	5.03 [0.08]
Davies *p*-value	[0.000]	[0.000]	[0.000]	[0.000]	[0.000]	[0.000]	[0.000]	[0.000]	[0.000]	[0.000]

**Note:** *** represents statistical significance at the 1% level. The values in brackets are *p*-values.

## Data Availability

Data published in this paper are available upon request.
